# Body composition among Sri Lankan infants by ^18^O dilution method and the validity of anthropometric equations to predict body fat against ^18^O dilution

**DOI:** 10.1186/s12887-015-0371-2

**Published:** 2015-05-06

**Authors:** Thushari Bandara, Manjula Hettiarachchi, Chandrani Liyanage, Sujeewa Amarasena, William Wai-Lun Wong

**Affiliations:** Faculty of Medicine, University of Ruhuna, Galle, Sri Lanka; USDA/ARS Children’s Nutrition Research Center, Department of Pediatrics, Baylor College of Medicine, Houston, TX USA

**Keywords:** Total body water, Fat mass, Fat free mass, Isotope ratio mass spectrometry, ^18^O dilution, Skinfold thickness, Mid upper-arm circumference

## Abstract

**Background:**

Body composition indicators provide a better guidance for growth and nutritional status of the infants. This study was designed to (1) measure the body composition of the Sri Lankan infants using a reference method, the ^18^O dilution method; (2) calculate the body fat content of the infants using published skinfold prediction equations; and (3) evaluate the applicability of the skinfold equations to predict body fat among Sri Lankan infants against the ^18^O dilution method.

**Methods:**

Twenty five healthy, exclusively breast-fed infants were randomly recruited at well-baby clinics, for this cross-sectional study. Body composition was measured using ^18^O dilution. Infant body weight, length, skinfold thicknesses and mid upper-arm circumference were measured using standard procedures. The Bland and Atlman pair-wise comparison method was used to evaluate the agreement of body fat generated using the anthropometric prediction equations against the ^18^O dilution values as the reference.

**Results:**

Mean (SD) body weight and length of the infants were 6.5 kg (0.9) and 64.7 cm (2.8) respectively. Mean total body water, fat free mass, fat mass and % fat mass as measured by ^18^O dilution method were 58.8% (5.0), 4.6 kg (0.8), 1.9 (0.5) and 29.5% (6.1). Total body water and fat free mass were significantly higher in boys when compared to girls. With the exception of three prediction equations (Bandana *et al.*, Goran *et al.* and Durnin and Wormsley), most of the other commonly used anthropometry-based prediction equations yielded a bias which was not constant but a function of the % fat mass.

**Conclusions:**

Body composition of Sri Lankan infants is comparable to the normative data available from the industrialized countries. Most of the commonly used anthropometric prediction equations generated a bias which varies with the size of the body fat. Only three prediction equations (Bandana, Goran, Durnin & Wormsley) yield a constant bias. The Durnin & Wormsely equation showed the smallest bias when compared to the ^18^O dilution values with the narrowest limits of agreement. Accuracy of some of the prediction equations is a function of gender.

**Electronic supplementary material:**

The online version of this article (doi:10.1186/s12887-015-0371-2) contains supplementary material, which is available to authorized users.

## Background

Body composition generally refers to the percentages of water, fat, bone and muscle in a human body. It is acknowledged that the assessment of body composition provides a much better gauge on the growth and nutritional status of an infant than weight and length due to the fact that the infants of similar weight, length and or weight for length have shown substantial differences in body composition [1]. These differences are based on their body composition phenotypes which mark fundamental metabolic differences as well as greater risks of non-communicable diseases later in life [2]. Thus the assessment of body composition is considered valuable in pediatric care.

Methods for the measurement of body composition have developed on the basis of two or four compartment models. The 2-compartment model considers the body consists of two chemically distinct compartments, namely fat mass (FM) and fat-free mass (FFM) whereas the 4-compartment model divides the mammalian body into four chemical groups: water, protein, ash or bone mineral and fat [3]. The 2-compartment model based on isotope dilution is considered one of the reference methods for body composition measurements and is most often used since the procedure is non-invasive and portable, so that it can be implemented anywhere to a large number of study subjects at the same time [1]. The present study was designed to measure the body composition of healthy, exclusively breastfed (EBF) Sri Lankan infants using the stable isotope ^18^O dilution method and by anthropometry and to evaluate the validity of commonly used anthropometric equations to predict body composition of Sri Lankan infants.

## Methods

### Study design

Due to difficulties of enrolling infants to take part in the very first stable isotope study in Sri Lanka, we adopted the purposive and convenient sampling technique without sample size consideration for this cross-sectional study. Twenty-five infants aged 4–6 months were randomly selected from well-baby clinics of the Medical Officer of Health (MOH) areas in Galle, Sri Lanka. All infants were exclusively breast fed from birth. Infants with any congenital anomalies, chronic diseases or any illnesses were excluded based on the infant’s previous medical histories, drug treatments and medical tests performed since birth and on the medical examination by the physician. No additional biological tests were done to exclude infants with anemia. The eligibility of the infants was checked by a brief demographic and health questionnaire prior to the enrollment. Study protocol was approved by the Ethical Review Committee, Faculty of Medicine, University of Ruhuna, Sri Lanka. Institutional approvals were obtained from the Ministry of Healthcare and Nutrition and from the selected MOH offices. Informed consent was obtained in writing from all mothers, after explaining the procedures and expected outcomes of the research.

### Body composition by the isotope dilution technique

Infants (nude) were weighed using a standard electrical scale [Seca 334 electronic baby scale (Medical) Seca Gmbh and Co, Germany] with a precision of 10 g. Recumbent length of the infants was measured using an infantometer (Raven equipment Ltd, England) to the nearest 0.1 cm. A pre-dose urine sample (2 mL) was collected from each infant by keeping sterile cotton wool inside the nappies of baby and expressing the urine from the cotton wool using a 20-mL syringe. An accurately measured (analytical balance, model HR-200, Japan, A&D company Ltd) dose of ^18^O water (10% ^18^O enrichment, from Sigma Aldrich) at 10 mg/kg body weight was administered orally to each infant using a sterile syringe without the needle. The dose syringe was rinsed twice with minimum volume of drinking water and fed to the infant in order to ensure that the infant received the entire isotope dose. Post dose urine samples were collected after 5 hours and on day 3. Urine samples were kept in water-tight tubes, frozen at − 20°C, stored and transported to the Gas-Isotope-Ratio Mass Spectrometry Laboratory at the USDA/ARS Children’s Nutrition Research Center at Baylor College of Medicine in Houston, Texas, USA for isotopic analyses.

### Analysis of the ^18^O enrichment

Samples were processed for stable ^18^O isotope ratio measurements by gas-isotope-ratio mass spectrometry according to the procedures developed and validated by Wong WW [4]. Briefly, 100 μl of sample was allowed to equilibrate with CO_2_ of known ^18^O content at 25°C for 10 h. The CO_2_ was then introduced into a VG SIRA-12 gas-isotope-ratio mass spectrometer system for stable oxygen isotope ratio measurement. The method was accurate to −0.32‰ (5.4 ppm) and reproducible to within 0.97‰ (2.8 ppm) based on analysis of ^18^O reference materials. These reference materials included the Vienna-Standard Mean Ocean Water, the Vienna-Standard Light Antarctic Precipitation, and the reference water samples enriched with ^18^O (IAEA 304A and 304B) [5]. The ^18^O content of these reference materials ranged from natural abundance to 500‰.

### Calculations

The basic principle for the determination of total body water (TBW) by isotope dilution technique is based on the fact that when a known amount of tracer (isotope) is added to a fixed volume of water and the isotope is allowed to be uniformly distributed within the volume of water, the original volume of water can be quantified based on the amount of tracer added and its relative concentration at equilibrium. The back extrapolation method which is considered more accurate for infants and young children [1] was used to generate the isotope concentration at time zero by plotting the log transformed tracer concentration at 5-hr and 3-day post dose against time. The intercept or the zero time intercept value for the straight line represents the tracer concentration at equilibrium [6,7]. The back extrapolation method rather than the equilibration method was used in this study because it normalized the effect of fluid intake, metabolic water, or fluid losses through urination, respiration and sweet on the body water compartment.

Isotope dilution space (NO) was calculated with the following equation:

$$ \begin{array}{l}\mathrm{NO}\ \left(\mathrm{kg}\right) = \Big[\mathrm{Dose}\ \mathrm{given}\ \mathrm{t}\mathrm{o}\ \mathrm{in}\mathrm{f}\mathrm{ant}\ \left(\mathrm{g}\right)\times \mathrm{Weight}\ \mathrm{o}\mathrm{f}\ \mathrm{water}\ \mathrm{used}\ \mathrm{in}\ \mathrm{t}\mathrm{he}\ \mathrm{dose}\ \mathrm{dilution}\ \left(\mathrm{g}\right){\times}^{18}\mathrm{O}\ \mathrm{enrichment}\ \mathrm{o}\mathrm{f}\ \mathrm{t}\mathrm{he}\ \\ {}\mathrm{dose}\ \mathrm{dilution}\left]/\right[\mathrm{Weight}\ \mathrm{o}\mathrm{f}\ \mathrm{dose}\ \mathrm{used}\ \mathrm{in}\ \mathrm{dose}\ \mathrm{dilution}\ \left(\mathrm{g}\right){\times}^{18}\mathrm{O}\ \mathrm{enrichment}\ \mathrm{at}\ \mathrm{t}\mathrm{ime}\ \mathrm{zero}\times 1000\Big]\end{array} $$

TBW was calculated using the relationship, TBW (kg) = NO (kg)/1.01 (the constant, 1.01, was used to adjust for the non-aqueous ^18^O exchange with tissues [8].

FFM was calculated using the relationship, FFM (kg) = TBW/H_LM,_ where H_LM_ is the age and sex specific reference values for lean tissue hydration [9]. FM was the difference between body weight and FFM.

### Determination of body fat by anthropometry

All anthropometric measurements were made according to the Anthropometric Standardization Reference Manual [10]. Triceps, biceps, subscapular and suprailliac skinfold thicknesses of all infants were measured on the left side of the body in duplicates, with a skinfold caliper (Crymych, Holtain Ltd, UK). All measurements were made on the same day when the infant underwent the ^18^O dilution test. Triceps skinfold thickness was the vertical fold measured at the posterior midline of the upper arm, halfway between the tip of the shoulder and tip of the elbow while the elbow remained in an extended and relaxed position. Biceps skinfold thickness was the vertical fold measured at the anterior midline of the upper arm, directly above the centre of the cubital fossa, at the same level as the triceps skinfold. Subscapular skinfold thickness was the oblique fold measured just below the bottom tip of the scapula. Suprailliac skinfold thickness was the slightly oblique fold which follows the natural diagonal line and was measured immediately superior to the iliac crest, in the mid-axillary line [11]. Mid upper-arm circumference (MUAC) was measured at the midpoint of the left upper arm (extended with the palm facing inwards) between the acromion process and the tip of the olecranon, using a plastic non-stretchable tape to the nearest millimeter [11].

Infant body fat was calculated using sixteen prediction equations [12-23] as shown in the Additional file 1.

### Statistical analysis

Demographic characteristics were tabulated as mean ± SD for continuous measures and as percentage for dichotomous measures. Body composition was expressed as kg and percentage of body weight. The association between infant body composition on demographic and maternal characteristics was assessed by Pearson Correlation using Statistical Package for Social Sciences (version 20.0, SPSS Inc., Chicago). The Bland and Altman pair-wise comparison method [24] was used to evaluate the agreement between the % FM predicted using the anthropometric equations and the % FM measured using the ^18^O dilution method as the reference by plotting the differences between the two measurements against the averages of the two measurements to make sure that the differences were well distributed around zero. If not, regression analysis was used to test the differences between the two methods and their average % FM values. If the slope was not significant, the relative bias [mean difference (MD) between methods] and the 95% limits of agreement (MD ± 2 SD of the difference) were computed. If the slope relating the differences and average % FM was significant, the 95% limits of agreement were estimated as 2 standard error of estimate (SEE) around the regression line at 20% and 45% FM which represented the minimum and maximum values among the infants.

## Results

Body composition was assessed in the term, healthy, EBF babies (n = 25), aged 4–6 months. One infant had an infection during the sample collection period and was on treatment. Therefore this infant was excluded from the sample. Infant characteristics and anthropometric measurements are presented in Table 1. Mean (SD) age of the infants was 4.5 months (0.8). Mean birth weight was 2.9 kg (0.6) with two girls having birth weights at 2.0 and 2.1 kg, respectively. These two baby girls were enrolled into the study because at the time of recruitment, they were 5.5 months and 4.25 months of age, respectively. Both of their body weights were above 6 kg at the time of recruitment suggesting satisfactory weight gain. There were 13 girls and 11 boys in the sample. Mean body weight and length were 6.5 kg (0.9) and 64.7 cm (2.8), respectively.

**Table 1:** **Infants’ characteristics (n = 24) and anthropometric measurements**

**Characteristic**	**Mean**	**SD**	**Range**
Age (months)	4.5	0.8	4.0–6.0
Birth weight (kg)	2.9	0.6	2.0–4.0
Weight (kg)	6.5	0.9	5.0–8.0
Length (cm*)*	64.7	2.8	60.3–69.8
Weight-for-age Z score	−0.41	1.5	−2.0–1.9
Height-for-age Z score	0.49	1.0	−1.4–2.4
Body mass index (kg/m^2^)	15.6	1.7	12.6–19.7
Skin-fold thicknesses (mm)			
Triceps	9.0	1.0	7.0–11.0
Biceps	7.4	1.1	5.0–9.0
Subscapular	8.5	1.0	7.0–11.0
Suprailliac	10.0	1.5	7.0–13.0
Mid upper arm circumference (cm)	14.4	1.0	12.0–16.0

The body composition of the infants as measured by ^18^O isotope dilution is presented in Table 2. Mean (SD) total body water of all infants was 58.8% (5.0). Mean FFM was 4.6 kg (0.8). Mean and % FM were 1.9 kg (0.5) and 29.5% (6.1), respectively. TBW and FFM were significantly higher in boys than in girls (p = 0.02 and p = 0.01), respectively.

**Table 2:** **Body composition of the infant by isotope dilution technique**

**Index**	**Girls (n = 13)**	**Boys (n = 11)**	**All infants (n = 24)**
Total body water (%)	56.7 (2.9)	61.3^*^ (5.9)	58.8 (5.0)
^18^O content (ppm)			
Baseline samples	1998.3 (0.7)	1998.3 (1.0)	1998.3 (0.8)
5-h samples	2190.1 (27.2)	2145.8 (17.0)	2168.8 (31.8)
3-d samples	2095.6 (15.8)	2080.5 (16.6)	2088.4 (17.6)
Fat free mass (kg)	4.3 (0.6)	5.0^**^ (0.8)	4.6 (0.8)
Fat mass (kg)	2.0 (0.5)	1.9 (0.6)	1.9 (0.5)
% Fat mass	31.3 (5.3)	27.3 (6.5)	29.5 (6.1)

The single and double asterisks represents the 2-sample *t*-test between the boys and the girls (^*^p = 0.02, ^**^p = 0.01). ^18^O content represents the ^18^O content of the baseline and post-dose urine samples collected from the infants.

Results are given as mean (SD).

Mean (SD) and range of the percentage FM of the infants as calculated using the 16 anthropometry based prediction equations are presented in Table 3. Equations of Hoffman et al. [14] and Durnin and Rahman [17] resulted in totally unphysiological values on Sri Lankan infants. The mean (SD) % FM of infants was 279.6 (27.1) according to the Hoffman equation and it was − 153.3 (7.9) according to the Durnin and Rahman equation. Therefore they were excluded from the tables. When compared to the mean % FM of all infants measured by the ^18^O dilution method, only the Bandana *et al.* [12] equation resulted in a higher value (33.8% *vs*. 29.5%). Percentage FM calculated by the other equations were lower (ranged from 16.8% – 25.6%) than the isotope dilution value.

**Table 3:** **Percentage fat mass of the infants by anthropometric prediction equations**

**Prediction equation**	**Mean**	**SD**	**Range**
Bandana *et. al.,* 2010 [12]	33.8	12.0	15.7–55.9
Shaikh & Dilip, 2004 [13]	20.5	1.6	17.6–24.0
Goran *et al*., 1996 [15]	19.6	4.0	9.4–26.6
Slaughter *et al.*, 1988 [16]	16.8	1.3	13.9–19.5
Slaughter *et al.*, 1988 [16]			
All female	20.3	1.7	17.5–23.8
White female	19.5	2.0	15.4–22.5
Black male	18.0	2.0	13.9–21.1
Brook, 1971 [18]	21.4	2.0	17.6–25.0
Yuan *et al*., 1987 [19]	20.6	0.9	18.5–22.5
Liu *et al*. [20]	16.8	1.3	13.9–19.5
Deurenberg *et al.,* 1990 [21]			
Equation 1	18.1	1.5	14.7–21.5
Equation 2	19.5	1.3	16.5–21.8
Durnin & Wormsley, 1974 [22]	25.6	1.3	23.6–27.5
Sloan *et al.,* 1962 [23]	16.8	0.8	15.9–18.3

The results of the Bland and Altman pair-wise comparison are summarized in Table 4. Pair-wise comparison was done for male and female separately. The first column identifies the skinfold equations used to predict % FM among the Sri Lankan infants. The second and third columns identify the bias between the two measurements and its standard deviation (SD). As shown in the table, none of the equations yielded perfect agreement or no bias but a systematic bias regardless of body fat. The third, fourth and fifth columns summarize the slope, the intercept and P value of the regression analysis performed between the differences and the average % FM values. For equations with no relationship (P > 0.05*)* between the differences and the average values, the lower and upper limits of agreement or 95% confidence interval between the two methods (skinfold equations *vs.*^18^O dilution) are shown as bias − 2SD and bias + 2SD, respectively. For equations showing significant relationship between the differences and the average values, the bias as well as the lower and upper limits of agreement varied depending on the % FM values. Since the minimum and maximum values for % FM among the Sri Lankan infants ranged between 20% and 45%, the limits of agreements for these equations were calculated at 20% and 45% FM for illustrational purposes.

**Table 4:** **Bland and Altman pair-wise comparison (all values in the table are expressed as percentage unit)**

**Equations**	**Bias**	**SD**	**Slope**	**Intercept**	**P**	**Bias-2SD**	**Bias + 2SD**	**LL at 20%**	**UL at 20%**	**LL at 45%**	**UL at 45%**
**For male**											
Bandana	17.84	9.63	−0.536	37.250	0.569	−1.42	37.11				
Shaikh	−5.49	6.68	−1.928	41.812	0.000			−10.11	16.61	−58.32	−31.60
Goran	−7.47	8.58	−0.706	9.139	0.363	−24.62	9.68				
Slaughter	−10.12	6.91	−1.934	32.850	0.000			−12.75	1.07	−61.11	−47.29
Slaughter (white)	−7.75	7.12	−1.837	35.248	0.002			−15.73	12.73	−61.67	−33.21
Slaughter (black)	−9.25	7.12	−1.837	32.369	0.002			−18.61	9.85	−64.54	−36.08
Brook	−4.59	6.94	−2.028	46.083	0.000			−8.37	19.41	−59.08	−31.30
Yuan	−6.50	6.76	−2.009	41.762	0.000			−11.93	15.09	−62.16	−35.14
Liu	−10.12	6.91	−1.935	32.850	0.000			6.17	7.99	−68.03	−40.37
Deurenberg (Equation 1)	−9.63	6.83	−1.980	34.845	0.000			−18.41	8.91	−67.91	−40.59
Deurenberg (Equation 2)	−8.45	6.80	−2.057	38.961	0.000			−15.78	11.40	−67.21	−40.03
**For female**											
Bandana	−7.20	6.83	0.108	−10.199	0.824	−20.85	6.45				
Shaikh	−11.99	5.29	−0.951	27.023	0.000			−2.57	18.59	−26.34	−5.18
Goran	−11.96	4.53	−0.538	1.690	0.112	−21.01	−2.91				
Slaughter	−14.76	4.90	−1.536	22.057	0.000			−18.45	1.13	−56.85	−37.27
Slaughter (all female)	−11.08	4.76	−1.284	22.078	0.000			−13.13	5.91	−45.24	−26.20
Brook	−11.07	4.97	−1.322	23.067	0.000			−13.32	6.57	−46.38	−26.49
Yuan	−10.90	5.04	−1.729	33.881	0.000			−10.78	9.38	−54.00	−33.85
Liu	−14.76	4.90	−1.536	22.057	0.000			−18.45	1.13	−56.85	−37.26
Deurenberge (Equation 1)	−12.90	5.10	−1.406	22.097	0.000			−16.20	4.18	−51.34	−30.96
Deurenberg (Equation 1)	−11.29	5.02	−1.573	29.170	0.000			−12.36	7.74	−51.70	−31.60
Durnnin & Wormsley	−5.79	5.01	−0.492	6.798	0.680	−15.81	4.23				
Sloan	−14.59	5.28	−1.839	29.654	0.000			−17.68	3.42	−63.67	−42.57

Descriptions and references for the each equation are illustrated in the additional pdf file [see Additional file 1]. Slope, slope of the linear regression analysis between the differences in %FM of the two methods and their average values; intercept, intercept of the linear regression analysis; P, p value of the regression analysis performed between the differences and the average %FM values; Bias-2SD and Bias + 2SD, 95% confidence level of the bias; LL, lower limit; UL, upper limit; 20%, the minimal %FM measured on our infants; 45%, the maximal %FM measured on our infants.

Among the prediction equations, only the % FM predicted by Bandana *et al.* equation [12] and the Goran *et al.* equation [15] showed no significant relationship between the differences and the average values when compared to the ^18^O dilution values for both male and female Sri Lankan infants. The equation by Durnin and Wormsley [22], which was developed specially for girls, also showed no significant relationship between the differences and average values among the female infants. The bias of the Bandana equation was higher in male infants when compared to female infants (17.8% *vs.* − 7.2%). The 95% limit of agreement for male infants also was higher than the female infants (SD, 9.63% for male *vs*. 6.83% for females). The bias of the Goran equation was higher for the female infants when compared to the male infants (−11.9% *vs.* -7.5%). However, the limits of agreement were narrower among the females (SD = 4.5%) than the males (SD = 8.5%). The Durnin and Wormesley equation produced the smallest bias for female infants (−5.8%) with a limit of agreement between − 15.8 and − 4.2%.

Unfortunately, all the other prediction equations showed significant relationship (P < 0.05) between the differences and the average values indicating that the difference would not remain constant as we have observed with the equations proposed by Bandana, Goran or Durnin & Wormesley but would change depending on the value of %FM. For example, with the Shaikh’s equation for baby boys, the equation would underestimate % FM by 5.49% (bias). However, the regression analysis between the differences and the average values revealed a significant slope (P = 0.000). Therefore, at 20% FM, the Shaikh’s equation could either underestimate %FM by 10.11% (LL at 20%) or overestimate % FM value by 16.61% (UL at 20%). At 45% FM, the Shaikh’s equation would underestimate %FM by 58.32% (LL at 45%) or by 31.60% (UL at 45%). Similar interpretations would apply to the other prediction equations showing significant relationship between the differences and the average values.

## Discussion

Body composition in early life plays a pivotal role in programming a wide array of health outcomes later in life, including obesity, hypertension, type 2 diabetes, cardiovascular diseases and stroke [25]. Measurement of body composition of infants is useful to evaluate the quantity and quality of the weight gain, monitor adequacy of physical growth and to study the effect of different nutritional regimens on the body composition [26]. However, limited data are available on the body composition of infants particularly among developing countries such as Sri Lanka. Many of the instruments developed for body composition assessment in humans have been designed for adults and modifications are usually required to be applied to children, particularly infants. For technical, theoretical as well as ethical reasons, most of the methodologies/instruments are not practical for the measurement of body composition in infants [27]. Therefore reliable measurement of infant body composition remains a technically challenging area [1].

Isotope dilution technique is a well established methodology for the measurement of human body composition [6,7,28]. This was our method of choice in the present study as it is considered the reference method for assessing TBW [1], and the procedure is non-invasive, safe and therefore suitable for pediatric use. Further, this is one of the body composition methods which can be used in non-urban and local settings [1]. To our knowledge this is the first study carried out in Sri Lanka to measure the body composition of EBF infants.

TBW denotes the water content of a human body. It makes up a significant portion of the human body, both by weight and by volume. Percentage of body water varies based on number of factors such as age, population, weight, gender and health conditions. Prediction of the infant TBW is useful in pediatric clinical practice in estimating fluid and energy requirements in parenteral nutrition, estimating pharmacological dosages and peritoneal dialysis dosages etc. [29]. In the present study the mean (SD) TBW of the infants (mean age of 4.5 months) was 58.8% of the body weight (5.0). It was 56.7% (2.9) in girls and 61.3% (5.9) in boys and the difference was significant (p = 0.02). Friis-Hensen *et al.* [30] measured the TBW of normal infants and children (n = 24) in USA by the deuterium dilution technique and reported that it ranged between 70% and 83% in newborns with a gradual reduction during the first 6 months of life. From 6 months to 11 years of age, the values varied between 53% and 63% with no correlation to age or sex. Fomon *et al.* [31] published the age and sex specific reference data for the body composition of children from birth to 10 years of age. Their data represent a variety of sources in the literature. The reference TBW for boys was 60.1%, 59.6% and 59.4% at 4, 5 and 6 months of life, respectively. For girls it was 59.6%, 58.8% and 58.4% respectively. According to normative body composition data by Butte *et al.* [9] on healthy, term infants in USA by the deuterium dilution method, the TBW of boys were 55.9% and 56.5% at 3 and 6 months, respectively. For girls the values were 55.4% and 55.3%, respectively. The TBW values of the 4.5-month-old Sri Lankan infants were well within the values reported by Fomons. Our values were higher when compared to the values reported by Butte on the 3-month-old USA infants. The difference could simply be due to the difference in hydration status between the Sri Lankan infants and the USA infants.

In the present study the mean (SD) % FM of all infants was 29.5% (6.1). It was 27.3% (6.5) in boys and 31.3% (5.3) in girls and there was no significant difference. According to Fomon *et. al* reference data [31], % FM for boys were 24.7%, 25.3% and 25.4% at 4, 5 and 6 months, respectively. The respective values for girls were 25.2%, 26.0% and 26.4%. According to Butte *et. al* normative data [9] % FM of boys at 3 and 6 months were 30.2% and 29.1%, respectively. The respective values for girls were 31.6 and 32.2%. Field *et. al* [32] also has published longitudinal body composition data in EBF infants (a multicenter study). According to them, the reference % FM for boys at 4, 5 and 6 months of age were 25.3%, 26.2% and 25.9%, respectively. The respective values for girls were 27.0%, 27.7% and 28.1%.

Validation of the body fat as measured by the anthropometric prediction equations with reference to isotope dilution, air displacement plethysmography or dual-energy X-ray absorptiometry has been reported from both developed and developing countries. However, only a few of these have been conducted on infants. Liu *et al*., compared the body fat of infants in Ireland, using skinfold thickness measurements and simultaneously with air displacement plethysmography [20]. Their study has produced reference values for % FM for infants at 8 weeks of age (mean: 21.07 and SD: 4.01). Furthermore, they concluded that the % FM calculated by skinfold measurements consistently underestimated the body fat. Our findings also support their conclusion as the equations they used produced the lowest % FM for Sri Lankan infants. Bandana *et al.,* developed prediction equations for % FM for Indian infants aged 6–24 months, based on skinfold thickness, mid-upper arm circumference, and age using deuterium dilution technique as the reference method [12]. The mean of the differences of paired values in % FM by the deuterium dilution and the developed prediction equations were zero and thus these equations have been recommended to be used on infants up to two years of age in the developing countries. Their equations did not show any significant relationship between the differences and the average values when compared to the ^18^O dilution values for both male and female Sri Lankan infants in the present study.

The ^18^O isotope dilution technique has gained a reputation to be more accurate and precise in contrast to the deuterium dilution method because ^18^O has less exchange with non-aqueous body constituents. Our ^18^O dilution data were corrected for the 1% non-aqueous exchange with the body tissues [8]. One of the limitations of the study is the small sample size due to recruitment difficulties in developing countries when stable isotopes are used.

## Conclusions

Body composition of Sri Lankan infants is similar to the normative data. Most of the commonly used skinfold prediction equations generated a bias which varies with the size of the body fat and therefore are not applicable for body fat measurements among Sri Lankan infants. The Hoffman equation definitely is not appropriate to be used to predict body fat among Sri Lankan infants because the bias was totally unphysiological. Only three prediction equations (Bandana *et.al*, Goran *et al.*, and Durnin & Wormsley) yield a constant bias. Durnin & Wormsely equation showed the smallest bias when compared to the ^18^O values with the narrowest limits of agreement. It was evident that the accuracy of some of these equations is a function of gender. Since most institutions in the developing countries will not be able to carry out the isotope dilution method, the skinfold prediction equations remain the most suitable and non-invasive methodology to measure body composition among infants. Therefore, we intend to generate a prediction equation using our ^18^O data and test its validity among Sri Lankan infants in future studies.

## Additional file

Additional file 1: A**nthropometric prediction equations used for the calculation of infant’s body fat.**

## Abbreviations

MOH: Medical Officer of Health
EBF: Exclusively breastfed
MUAC: Mid upper arm circumference
TBW: Total body water

## References

[1] International Atomic Energy Agency (2013). IAEA human health series No.22. Body composition assessment from birth to two years of age.

[2] Kensara OA, Wooton SA, Phillips DI, Patel M, Hoffman DJ, Jackson AA (2006). Substrate energy metabolism and metabolic risk factors for cardiovascular disease in relation to fetal growth and adult body composition. Am J Physiol Endocrinol Metab.

[3] Keys A, Brozek J (1953). Body fat in adult men. Physiol Rev.

[4] Wong WW, Lee LS, Klein PD (1987). Deuterium and oxygen-18 measurements on microliter samples of urine, plasma, saliva, and human milk. Am J Clin Nutr.

[5] Wong WW, Klein PD, Parr RM, Clements SA (1993). Interlaboratory analysis of reference water samples enriched with deuterium and oxygen-18. Appl Radiat Isot.

[6] International Atomic Energy Agency (2011). Introduction to body composition assessment using the deuterium dilution technique with analysis of saliva samples by fourier transform infrared spectrometry, IAEA Human Health Series No. 12.

[7] International Atomic Energy Agency (2011). Introduction to body composition assessment using the deuterium dilution technique with analysis of urine samples by isotope ratio mass spectrometry, IAEA Human Health Series No. 13.

[8] Schoeller DA, Santen E, Peterson DW, Dietz W, Jaspan J, Klein PD (1980). Total body water measurement in humans with ^18^O and ^2^H labeled water. Am J Clin Nutri.

[9] Butte NF, Hopkinson JM, Wong WW, Smith EO, Ellis KJ (2000). Body composition during the first 2 years of life: an updated reference. Pediatr Res.

[10] Lohman TG, Roche AF, Martorell R (1988). Anthropometric standardization reference manual.

[11] Gibson RS (1990). Principles of nutritional assessment.

[12] Sen B, Bose K, Shaikh S, Mahalanabis D (2010). Prediction equations for body-fat percentage in Indian infants and young children using skinfold thickness and mid-arm circumference. J Health Popul Nutr.

[13] Shaikh S, Mahalanabis D (2004). Empirically derived new equations for calculating body fat percentage based on skinfold thickness and midarm circumference in preschool Indian children. Am J Human Biol.

[14] Hoffman DJ, Toro-Ramos T, Sawaya AL, Roberts SB, Rondo P (2012). Estimating total body fat using a skinfold prediction equation in Brazilian children. Ann Human Biol.

[15] Goran MI, Driscoll P, Johnson R, Nagy TR, Hunter G (1996). Cross calibration of body-composition techniques against dual-energy Xray absorptiometry in young children. Am J Clin Nutr.

[16] Slaughter MH, Lohman TG, Boileau RA, Horswill CA, Stillman RJ, Van Loan MD (1988). Skinfold equations for estimation of body fatness in children and youth. Hum Biol.

[17] Durnin JVGA, Rahaman MM (1967). The assessment of the amount of fat in the human body from measurements of skinfold thickness. Br J Nutr.

[18] Brook CGD (1971). Determination of body composition of children from skinfold measurements. Arch Dis Child.

[19] Yuan T, Xin C, Sun Y (1987). Study of body composition and obesity definition of children and adolescents. Chinese J Prevent Med.

[20] Liu NH, Hourihane JO, Kenny L, Kiely M, Irvine AD, Murray DM (2010). Comparison of body fat estimation using skinfold thickness measurement and simultaneous air displacement plethysmography at 8 weeks. Pediatr Res.

[21] Deurenberg P, Pieters JJL, Hautvast JGAJ (1990). The assessment of the body fat percentage by skinfold thickness measurements in childhood and young adolescence. Br J Nutr.

[22] Durnin JVGA, Womersley J (1974). Body fat assessed from total body density and its estimation from skinfold thickness: measurements on 481 men and women aged from 16–72 years. Br J Nutr.

[23] Sloan AW, Burt JJ, Blyth CS (1962). Estimation of body fat in young women. J Appl Physiol.

[24] Altman DG, Bland JM (1983). Measurement in Medicine: the analysis of method comparison studies. The Statistician.

[25] Wells JC, Chomtho S, Fewtrell MS (2007). Programming of body composition by early growth and nutrition. Proc Nutr Soc.

[26] Schmelzle HR, Fusch C (2002). Body fat in neonates and young infants: validation of skinfold thickness versus dual-energy X-ray absorptiometry1–3. Am J Clin Nutr.

[27] Ellis KJ, Yao M, Shypailo RJ, Urlando A, Wong WW, Heird WC (2007). Body-composition assessment in infancy: air-displacement plethysmography compared with a reference 4-compartment model. Am J Clin Nutr.

[28] International Atomic Anergy agency (2009). Assessment of body composition and total energy expenditure in humans using stable isotope techniques, IAEA Human Health Series No. 3.

[29] Wells JCK, Fewtrell MS, Davies PSW, Williams JE, Coward WA, Cole TJ (2005). Prediction of total body water in infants and children. Arch Dis Child.

[30] Friis-Hansen BJ, Holiday M, Stapleton T, Wallagce WM (1951). Total body water in children. Pediatrics.

[31] Fomon SJ, Haschke F, Ziegler EE, Nelson SE (1982). Body composition of reference children from birth to age 10 years. Am J Clin Nutr.

[32] Fields DA, Janet M. Gilchrist, Patrick M. Catalano, Giannì ML, et al. Longitudinal body composition data in exclusively breast-fed infants: A multicenter study. Obesity. 2011, doi:10.1038/oby.2011.11. 10.1038/oby.2011.11 21311509

