# Supplementary material for: Body composition among Sri Lankan infants by 18O dilution method and the validity of anthropometric equations to predict body fat against 18O dilution
Source: BMC Pediatr. 2015 May 6;15:52. doi: 10.1186/s12887-015-0371-2 (PMC4428108; doi:10.1186/s12887-015-0371-2)
Supplement: Additional file 1: — Anthropometric prediction equations used for the calculation of infant’s body fat. [file 12887_2015_371_MOESM1_ESM.docx]

**Additional file 1**

**Anthropometric prediction equations used for the calculation of infant’s body fat**

| Equation code & Reference number | Equations^*^ | Race | Age |
| --- | --- | --- | --- |
| Bandana *et al.,* 2010 [12] | For girls: %FM = -69.26 + 5.76×B - 0.33×T^2^ + 5.40×M + 0.01×A^2^  For boys: %FM = -8.75 + 3.73×B + 2.57×SI | Indians | 6-24 months |
| Shaikh & Dilip, 2004 [13] | For boys: %FM = 5.304 + 0.269xT + 0.50xSS + 0.685xM - 0.063xA  For girls: %FM = 7.017 – 0.053xT + 0.201xSS + 0.765xM + 0.052xA | Indians  & South Asian children | Pre-school children |
| Hoffman *et al*., 2012 [14] | FM (kg) = 6.371 + 0.488xW + 0.128xT (11.138xH + 0.645xGender – 0.188xA^*^)  Gender as male=0 and female=1 | Brazilian Children | 9.8 years |
| Goran *et al*., 1996 [15] | For both genders:  FM (kg) = 0.18xW + 0.23xSI + 0.13xT - 3.0 | Burlington | Young children  4-10 years |
| Slaughter *et al.*, 1988 [16] | Boys : FM (kg) = W x [(1.21 x SUMTRSS - 0.008x(SUMTRSS)^2^ -1.7)/100]  Girls : FM (kg) = W x [(1.33 x SUMTRSS - 0.013x(SUMTRSS)^2^ – 2.5)/100] |  | Pre-pubertal |
| Slaughter *et al.*, 1988 [16] | White males: %FM = 1.21 × (T+SS) – 0.008 × (T+SS) - 1.7 | White male | Pre-pubertal |
| Slaughter *et al.*, 1988 [16] | Black males: %FM = 1.21 × (T+SS) – 0.008 × (T+SS) - 3.2 | Black male | Pre-pubertal |
| Slaughter *et al.*, 1988 [16] | All females: %FM = 1.33 × (T+SS) – 0.013 × (T+SS) - 2.5 |  | All female |
| Durnin & Rahaman, 1967 [17] | BD: Males = (1.1533–0.0643) × log (B+T+SS+SI)  Females = (1.1369–0.0598) × log (B+T+SS+SI)  Conversation of BD to %FM = 4.95/ BD - 4.5 × 100 |  | For adolescents |
| Brook, 1971 [18] | BD: Male = 1.1690 - 0. 0788 x log (B+T+SS+SI)  Female = 1.2063 - 0.0999 x log (B+T+SS+SI)  Conversation of BD to %FM = 4.95/ BD - 4.5 × 100 | UK  children | 1-11 years (pre-pubertal) |
| Yuan *et al*., 1987 [19] | %FM: Males = 9.0870 + 0.6616 × (T+SS)  Females = 11.2657 + 0.5311 × (T+SS) | Chinese | 10-12 years |
| Liu *et al*. [20] | %FM: Male = 1.21 x T+SS-0.008 x (T+SS)^2^-1.7  Female = 1.33 x T+SS-0.013 x (T+SS)^2^-2.5 | Ireland | Infants (8 wks old) |
| Deurenberg *et al.,* 1990 [21]  Equation 1 | %FM: Boys Prepubertal = -14.61 + 26.51 × log (B+T)  Girls Prepubertal = -16.84 + 29.3 × log (B+T) | Netherland | 7-20 years |
| Deurenberg *et al.,* 1990 [21]  Equation 2 | %FM: Boys Prepubertal = -22.23 + 26.56 × log (B+T+SS+SI)  Girls Prepubertal = -25.87 + 29.85 × log (B+T+SS+SI) | Netherland | 7-20 years |
| Durnin & Wormsley, 1974 [22] | %FM: Female = [(4.95/(1.1509-0.0715xlogS_1_)-4.5] x 100 | UK | 16-72 years old |
| Sloan *et al.,* 1962 [23] | % FM: Female = [4.95/(1.0764 – 0.00081xSI – 0.00088xT) - 4.5)] x 100 |  | Young female |

^*^All equations are for healthy individuals. %FM, Percentage fat mass; FM, fat mass; B, biceps skinfold thickness; T, triceps skinfold thickness; SI, suprailiac skinfold thickness; SS, subscapular skinfold thickness (all in mm); M, mid-arm circumference in cm; A, age in months; A^*^, age in years; W, weight (kg); H, height (m); SUMTRSS, sum of the triceps and subscapular skin fold thicknesses; BD, body density (kg/L); S_1_, sum of T, B, SS and SI skin fold thickness
